# Severe Urinary Retention Resulting in Extreme Post-obstructive Diuresis and Decompressive Hematuria

**DOI:** 10.7759/cureus.29626

**Published:** 2022-09-26

**Authors:** Jeanette K Klamfoth, Kathryn M Burtson

**Affiliations:** 1 Internal Medicine, Wright State University Boonshoft School of Medicine, Dayton, USA; 2 Internal Medicine, Wright-Patterson Air Force Base Medical Center, Dayton, USA

**Keywords:** obstructive hydronephrosis, acute kidney failure, gross hematuria, bph, chronic urinary retention, decompressive hematuria, post-obstructive diuresis

## Abstract

Post-obstructive diuresis and decompressive hematuria are rare but potentially serious complications of severe urinary retention. This is a case report of a 73-year-old patient with undiagnosed severe large-volume urinary retention who developed extreme cases of both complications after presenting with progressive weight gain, lower extremity edema, worsening dyspnea, and new-onset urinary incontinence. Upon further evaluation, the patient was determined to have acute renal failure, bilateral hydroureteronephrosis, a severely distended urinary bladder, and an enlarged prostate. Foley catheterization produced 5.9 L of urinary output with initial placement. He required prolonged hospitalization for hemodynamic monitoring requiring fluid resuscitation, serial electrolyte monitoring requiring repletion, acute blood loss anemia requiring four units of red blood cell transfusion, continuous bladder irrigation, three cystoscopies under anesthesia, and intravesicular fibrinolysis inhibitor instillations. This case illustrates an extreme severity of post-obstructive diuresis and decompressive hematuria in chronic urinary retention that has not been previously described in the literature.

## Introduction

Benign prostatic hyperplasia (BPH) affects three of four men over the age of 70 and is the most common benign neoplasm in American men [1]. Pathologically, it occurs as a result of the proliferation of epithelial and stromal cells in the prostate gland. Clinically, it is characterized by varying symptoms of urinary frequency, urinary urgency, nocturia, incomplete emptying of the bladder, urinary hesitancy, urinary incontinence, and weak urinary stream [1-2]. Urinary retention represents the final symptomatic stage of BPH and is associated with more severe symptoms [1]. Chronic urinary retention is the non-painful distention of the bladder with a chronically high post-void residual that is palpable or percussive in the presence or absence of incontinence or impaired renal function, as defined by The International Continence Society [3]. While there is no defined normal post-void residual volume, the commonly accepted value in chronic urinary retention is greater than 800 mL; however, patients often present with 1000 to 1500 mL [4-9]. Given the progressive and non-painful nature of this condition, it is not uncommon for large residual volumes to appear incidentally on radiologic imaging or for complications to arise insidiously [2]. The most common complications include overflow incontinence, recurrent urinary tract infections, hydronephrosis, edema, and acute or chronic renal failure [3-8]. More serious complications include post-obstructive diuresis and decompressive hematuria.

Post-obstructive diuresis is the polyuric state exceeding 200 mL/hr for two consecutive hours or 3 L in 24 hours, resulting in copious amounts of salt and water elimination and possible impairment of urinary concentration after relief of a long-standing bilateral ureteral obstruction [4-5,10]. Bishop estimated that post-obstructive diuresis occurred in 52% of patients with chronic urinary retention [7]; however, the reported incidence varies greatly and is highly dependent on the post-void bladder volume prior to decompression [11].

The withdrawal of high intravesicular pressure in a patient with a significantly distended bladder as in the case of chronic urinary retention can result in hemorrhage into the lumens of the urinary tract, resulting in decompressive hematuria [6,12]. In a review of 300 cases of chronic urinary retention, Glahn et al. estimated the incidence of decompressive hematuria to be 16% [13]. Most often when decompressive hematuria does occur it self-resolves within 24-48 hours, and hematuria requiring transfusion is much rarer [9,11].

This case demonstrates the complications of decompressive hematuria and post-obstructive diuresis to an exceedingly rare severity with diuresis causing hypovolemic shock and hematuria requiring blood transfusion. It is also amongst the largest recorded post-void residual, as found through an in-depth literature review, with a volume measuring 5.9 L.

## Case presentation

The patient was a 73-year-old gentleman who presented with a chief complaint of progressive weight gain, lower extremity edema, worsening dyspnea, and new-onset urinary incontinence. Two months prior, the patient underwent an echocardiogram due to his lower extremity edema and was diagnosed with heart failure with preserved ejection fraction and grade 1 diastolic dysfunction, for which he was started on furosemide. His urinary incontinence started after initiation of diuretic medication without improvement in weight gain or lower extremity edema. He returned due to the persistence of his symptoms.

**Figure 1:**
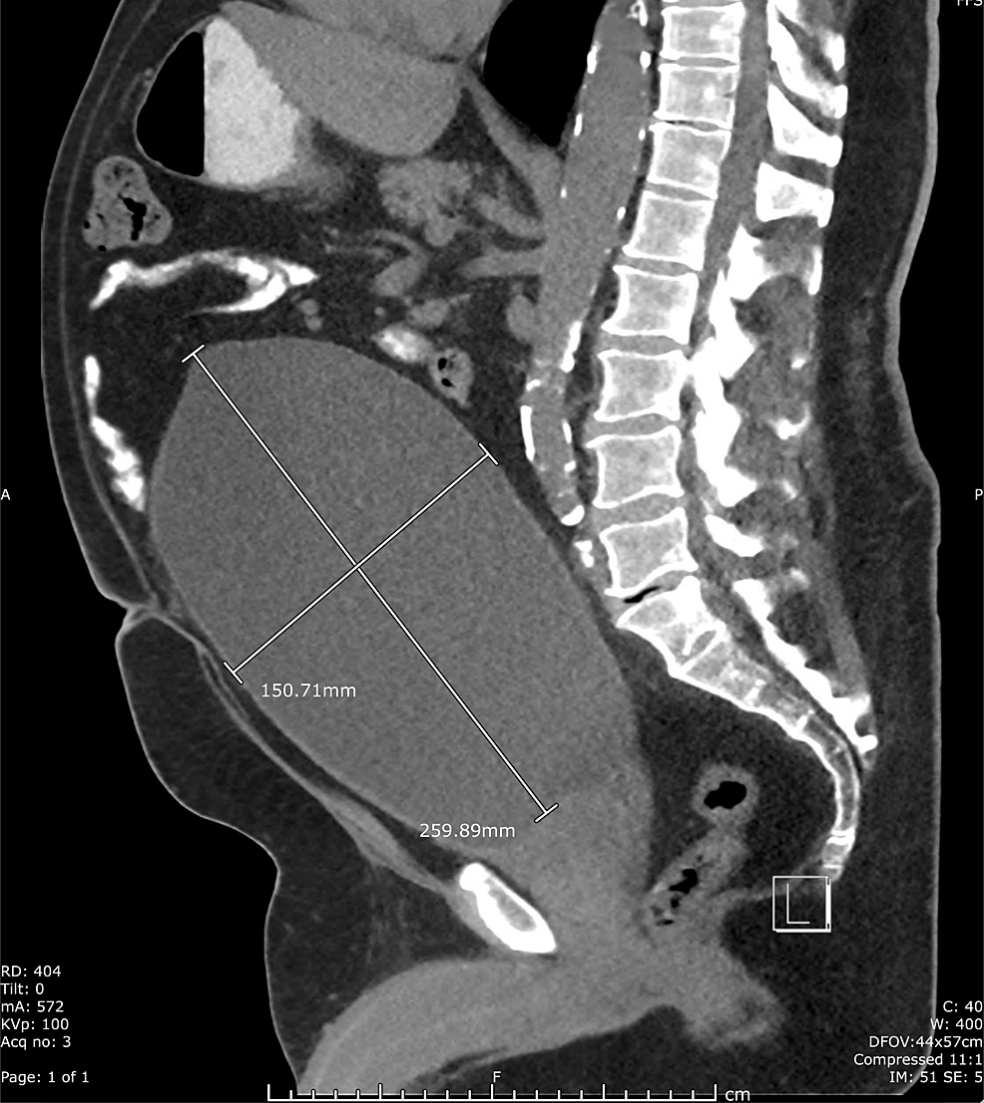
Sagittal view of the CT abdomen and pelvis with severe urinary bladder distention. The urinary bladder measures 259.89 mm by 150.71 mm.

**Figure 2:**
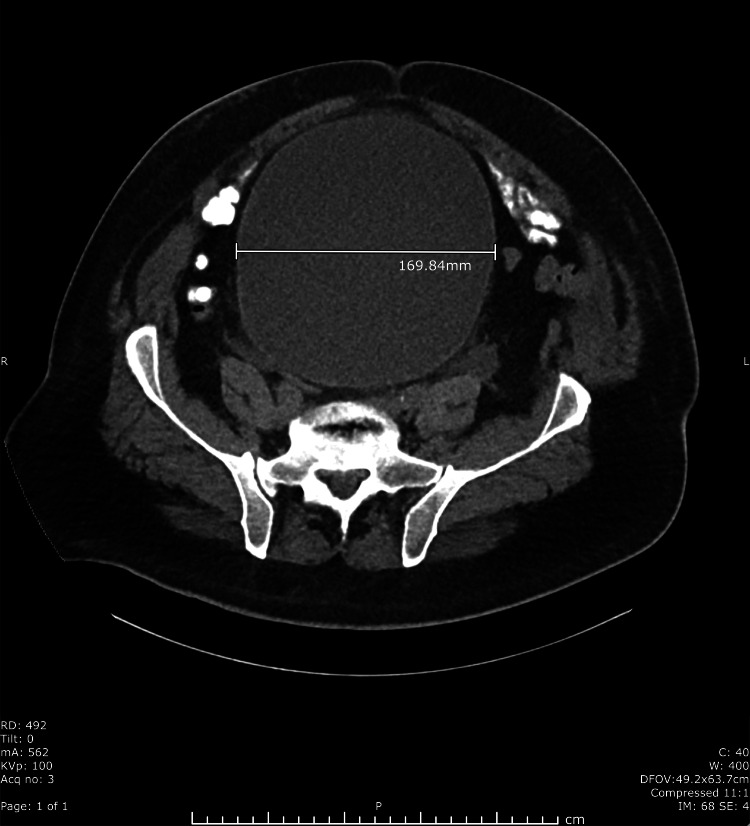
Axial view of CT abdomen and pelvis with severe urinary retention. The urinary bladder measures 169.84 mm in diameter.

His admission labs were notable for creatinine of 2.68 mg/dL from a baseline of 1.0 mg/dL. The timeline for the decline in kidney function correlated with the new heart failure diagnosis and initiation of the diuretic. A bedside bladder scanner was unable to detect a bladder volume due to enlarged size and abdominal edema. A computed tomography scan of the abdomen and pelvis was remarkable for significant distention of the urinary bladder, measuring 26 x 15 x 17 cm, with secondary severe bilateral hydroureteronephrosis and prostate gland enlargement [Figures 1, 2].

A foley catheter was placed by nursing without complication or significant trauma. His urinary output was 5.9 L with initial catheter placement and rapid bladder decompression. He became acutely hypotensive and was responsive to IV fluids without vasopressor support. He subsequently developed profound hematuria within the first hour after decompression of the bladder. His hematuria and acute blood loss anemia were treated with a transfusion of four units of packed red blood cells, continuous bladder irrigation, three cystoscopies under anesthesia, and aminocaproic acid instillations over the following eight days. The multiple cystoscopies performed could not identify the origin of bleeding for fulguration and were rather instrumental in clot evacuation for continued bladder irrigation. Over the course of his admission, the patient required intermittent potassium and magnesium repletion but did not experience extreme electrolyte derangements. His creatinine gradually improved to 1.1 mg/dL with post-renal obstruction as the etiology of the acute kidney injury. In addition, his bilateral lower extremity edema resolved due to post-obstructive diuresis. The patient was discharged home with an indwelling foley catheter and he was eventually transitioned to intermittent catheterization at outpatient urology follow-up. With the management of his urinary retention, he has not since required diuretic medications to maintain his weight or lower extremity edema.

## Discussion

The objective of presenting this case is to highlight the potential severity of post-obstructive diuresis and decompressive hematuria in the setting of chronic urinary retention secondary to BPH. Many inpatient hospitalizations require foley catheter insertion for medical management and this case begs attention for consideration of decompressive hematuria and post-obstructive diuresis as life-threatening complications following catheterization in addition to the infectious and traumatic risks associated with catheterization.

The cause of post-obstructive diuresis is multifactorial, including drainage of retained water, sodium, and urea, the impaired ability of the renal tubules to concentrate the urine due to loss of corticomedullary concentration gradients, circulation of atrial natriuretic peptide and similar hormones, and iatrogenic repletion of intravenous fluids [5]. While most patients do not experience significant consequences from post-obstructive diuresis, they are at risk for severe dehydration, hypovolemic shock, hypo- or hypernatremia, hypokalemia, hypomagnesemia, metabolic acidosis, and death [4]. Given this, they should be monitored closely with hemodynamic monitoring, strict intake and output, and serum and urinary electrolytes every six to 12 hours to appropriately replete intravenous fluids as well as sodium, potassium, and magnesium [4-5]. Most patients have improvement, if not full recovery, of renal function after decompression of the bladder and diuresis. However, if intrarenal pressures were prolonged, chronic renal failure may persist due to the loss of nephrons [7]. Additionally, the edema secondary to either renal impairment or pelvic venous compression resolves in most patients after decompression [7]. Our patient met the definition for post-obstructive diuresis with a urinary output of 5.9 L on initial catheterization. Furthermore, volumes of 3-4 L have been described in exceedingly rare cases of urinary retention; however, none were identified in an in-depth literature review to be nearing 6 L in volume [5-10]. He did develop hypovolemic shock secondary to rapid diuresis but fortunately did not develop severe electrolyte derangements. Additionally, our patient did have complete recovery of his renal function as well as resolution of his lower extremity edema.

The theorized mechanism of decompressive hematuria is that there is hyperemia to the large veins in the bladder mucosa that become grossly distended as a result of sudden pressure differences with decompression resulting in rupture of these vessels and hemorrhage into the urinary lumens [5,6,9]. It was because of this hypothesis that urologists initially believed that slow decompression of the bladder would limit hematuria. However, in practice, it has been determined that both gradual and rapid decompression were accompanied by bleeding [4-6,9,11-13]. Additionally, gradual decompression actually increased the risk of infection, was far more labor-intensive, was much more difficult to achieve, and overall did not lower the morbidity or mortality [5,11,12]. While most cases of decompressive hematuria are mild and usually self-resolve, it is associated with risks of acute blood loss anemia, hemorrhagic shock, and death. If hematuria develops, patients should be monitored closely for hemodynamic instability and anemia. Exceptionally rare cases have required bladder irrigation and blood transfusion [5]. Our patient experienced severe prolonged hematuria and required multiple transfusions of packed red blood cells, continuous bladder irrigation, multiple cystoscopies, and intravesicular fibrinolysis inhibitor instillations to control bleeding, an extent of severity that has not been previously described in the literature for decompressive hematuria.

In the majority of patients, post-obstructive diuresis and decompressive hematuria resolve spontaneously; however, some severe cases, as in our patient, may require aggressive management. Early detection of BPH and prevention of chronic urinary retention in the primary care setting should play a major role in reducing the risk of these serious complications. In the randomized clinical trial, Medical Therapy of Prostatic Symptoms (MTOPS) study, in 2003, alpha-blockers and 5-alpha-reductase inhibitors used in combination were almost twice as effective as monotherapy in preventing clinical progression of acute urinary retention, incontinence, renal insufficiency, or recurrent urinary tract infection as well as decreasing need for surgical intervention [1,14]. After a diagnosis of chronic urinary retention is made, scheduled micturition or self-catheterization is the mainstay for achieving near complete emptying of the bladder and is required to prevent the recurrence of large volume retention [5,6]. Surgical intervention may also be considered by a urologist as a means of long-term management, which is not discussed here.

## Conclusions

Post-obstructive diuresis and decompressive hematuria are rare but potentially lethal complications that can occur in the relief of severe urinary retention. The objective of this case is to exemplify the severity of these potential complications and educate healthcare professionals on these diagnoses. Post-obstructive diuresis and decompressive hematuria should always be considered before foley catheter placement. In the event of these complications, patients require strict monitoring of vital signs, hemoglobin levels, fluid status, serum electrolyte levels, and involvement of urology during hospitalization for treatment and stabilization.
